# TTTS Staging Updates: Balancing Evidence, Access, and Patient Care

**DOI:** 10.1002/pmf2.70112

**Published:** 2025-09-16

**Authors:** Jessian L. Munoz, Alireza A. Shamshirsaz

**Affiliations:** ^1^ Department of Obstetrics and Gynecology Baylor College of Medicine and Texas Children's Fetal Center Houston Texas USA; ^2^ Fetal Care and Surgery Center Boston Children's Hospital Harvard Medical School Boston Massachusetts USA

By design, Fetal Centers serve as tertiary referral sites for patients with complex fetal anomalies after early identification by Maternal Fetal Medicine subspecialists or Ob/GYN specialists [1]. This process inherently requires patient travel to the Fetal Center, which may introduce additional barriers to care such as financial resources, social support, and employment limitations, as well as psychological burden. Facilitating simple, yet effective, guidelines for the early identification and efficient referral process is essential, thus balancing the potential impact to patients while maximizing benefits to the pregnancy. The Delphi consensus study performed aims at further refining the 1999 Quintero Staging for Twin‐to‐Twin transfusion syndrome [2]. The significant implications for identification and referral should be considered.

Fetoscopic laser photocoagulation of monochorionic twin communicating vessels is currently recommended for those with TTTS Quintero Stage II and greater. In select cases of TTTS Quintero Stage I disease, such as maternal symptoms or short cervix, laser surgery may be considered. Uniform use of laser for TTTS Stage I is not recommended and this is supported by a randomized controlled trial, which showed that 41% had no progression and overall survival was equivalent between those randomized to laser surgery versus expectant management (77% vs. 78%) [3]. This Delphi consensus study defined “pre‐TTTS” as a precursor to TTTS and recommended increased surveillance in this setting. There exists a significant paucity of data to support both this designation and recommendation. In the event of disease progression, the aforementioned RCT showed stability or regression in a large portion of well‐characterized cases. A 2024 report on isolated oligohydramnios or polyhydramnios in the setting of MCDA twins did show 41% subsequently developed TTTS, yet the average latency period from ultrasound to TTTS diagnosis was 12 and 30 days (for isolated polyhydramnios and oligohydramnios, respectively). Subsequent latency from ultrasound to laser procedure was 101 and 104 days, respectively [4]. Increased weekly surveillance, as recommended, needs also to be considered in the social context of patient access, availability, maternal anxiety, and resource utilization.

Further inclusion of an “atypical” TTTS in which recipient fetuses exhibit cardiovascular changes without the initial presentation of TTTS (oligo‐/polyhydramnios) creates an additional designation without evidence‐based recommendations for diagnosis or management. Based on the current understanding of the pathophysiology of TTTS, optimization of potential pathophysiology, interventions, and implications remains complex and unknown. As proposed, these cases would be identified by echocardiogram without evidence of TTTS. Echocardiogram is often performed between 18 and 22 weeks and is recommended for monochorionic gestations [5]. TTTS staging systems, which have historically included echocardiogram in the setting of Stage III TTTS, have not been widely accepted [6, 7]. While echocardiogram access has increased over time, significant limitations exist in widespread usage, particularly in rural areas with few specialists [8].

This study opens the doors to further research in the field of fetal intervention and further characterization of the underlying pathophysiology of TTTS. Understanding the etiology and progression of TTTS is essential for the development of interventions and recommendations. Yet, patient access and simplicity of screening systems should remain at the forefront of patient care.

## References

[1] Chon, A. H. , A. J. H. Kim , R. Sohaey , L. Pereira , A. B. Caughey , A. C. Hermesch , A. A. Shamshirsaz , et al. 2025. “The Process of Developing a Comprehensive Maternal‐Fetal Surgery Center.” American Journal of Obstetrics & Gynecology MFM 7(1): 101557. 39580116 10.1016/j.ajogmf.2024.101557

[2] Quintero, R. A. , W. J. Morales , M. H. Allen , P. W. Bornick , P. K. Johnson , and M. Kruger . 1999. “Staging of Twin‐Twin Transfusion Syndrome.” Journal of Perinatology 19(8 Pt 1): 550–5. 10645517 10.1038/sj.jp.7200292

[3] Stirnemann, J. , F. Slaghekke , N. Khalek , N. Winer , A. Johnson , L. Lewi , M. Massoud , et al. 2021. “Intrauterine Fetoscopic Laser Surgery versus Expectant Management in Stage 1 Twin‐to‐Twin Transfusion Syndrome: An International Randomized Trial.” American Journal of Obstetrics and Gynecology 224(5): 528.e1–12. 10.1016/j.ajog.2020.11.031 33248135

[4] Buskmiller, C. , L. D. Chabolla , L. A. Radilla , D. Chilukuri , D. A. Dominguez , J. L. Munoz , A. A. Nassr , M. S. Cortes , M. A. Belfort , and R. V. Donepudi . 2025. “Adverse Outcomes in Monochorionic Twins With Amniotic Fluid Abnormalities Without TTTS: A Case‐Control Study.” European Journal of Obstetrics, Gynecology, and Reproductive Biology 306: 132–8. 39826275 10.1016/j.ejogrb.2025.01.021

[5] Patient Safety and Quality Committee, Society for Maternal‐Fetal Medicine, I. A. Hoskins , and C. A. Combs . 2020. “Society for Maternal‐Fetal Medicine Special Statement: Updated Checklists for Management of Monochorionic Twin Pregnancy.” American Journal of Obstetrics and Gynecology 223(5): B16–20. 10.1016/j.ajog.2020.08.066 32861686

[6] Rychik, J. , Z. Tian , M. Bebbington , F. Xu , M. McCann , S. Mann , R. D. Wilson , and M. P. Johnson . 2007. “The Twin‐Twin Transfusion Syndrome: Spectrum of Cardiovascular Abnormality and Development of a Cardiovascular Score To Assess Severity of Disease.” American Journal of Obstetrics and Gynecology 197(4): 392.e1–8. 10.1016/j.ajog.2007.06.055 17904973

[7] Villa, C. R. , M. Habli , J. K. Votava‐Smith , J. F. Cnota , F. Y. Lim , A. A. Divanovic , Y. Wang , and E. C. Michelfelder . 2014.“Assessment of Fetal Cardiomyopathy in Early‐Stage Twin‐Twin Transfusion Syndrome: Comparison Between Commonly Reported Cardiovascular Assessment Scores.” Ultrasound in Obstetrics & Gynecology 43(6): 646–51. 24151229 10.1002/uog.13231

[8] Woo, J. L. , S. Burton , T. Iyengar , A. Sivakumar , S. Spiewak , R. Wakulski , W. A. Grobman , et al. 2024. “Patient‐Reported Barriers to Prenatal Diagnosis of Congenital Heart Defects: A Mixed‐Methods Study.” Prenatal Diagnosis 44(1): 57–67. 38108462 10.1002/pd.6481

